# Flexible nanopillar-based immunoelectrochemical biosensor for noninvasive detection of Amyloid beta

**DOI:** 10.1186/s40580-020-00239-2

**Published:** 2020-09-01

**Authors:** Yoo Min Park, Junhyoung Ahn, Young Sun Choi, Jae-Min Jeong, Seok Jae Lee, Jae Jong Lee, Bong Gill Choi, Kyoung G. Lee

**Affiliations:** 1 Division of Nano-Bio Sensor/Chip Development, National NanoFab Center (NNFC), Daejeon, 34141 Republic of Korea; 2 Department of Nano Manufacturing Technology, Nano-Convergence Mechanical Systems Research Division, Korea Institute of Machinery & Materials (KIMM), Daejeon, 34103 Republic of Korea; 3 Department of Nanoengineering, University of California San Diego, La Jolla, CA 92093 USA; 4 Department of Chemical Engineering, Kangwon National University, Samcheok, 25913 Republic of Korea

**Keywords:** Noninvasive, Flexible, Nanopillar array, Immunoelectrochemical sensor, Alzheimer’s disease

## Abstract

The noninvasive early detection of biomarkers for Alzheimer’s disease (AD) is essential for the development of specific treatment strategies. This paper proposes an advanced method for fabricating highly ordered and flexible nanopillar-based electrochemical biosensors by the combination of soft/photolithography and metal evaporation. The nanopillar array (NPA) exhibits high surface area containing 1500 nm height and 500 nm diameter with 3:1 ratio. In regard with physical properties of polyurethane (PU) substrate, the developed NPA is sustainable and durable to external pressure such as bending and twisting. To manipulate the NPA surface to biocompatible, the gold was uniformly deposited on the PU substrate. The thiol chemistry which is stably modified on the gold surface as a form of self-assembled monolayer was employed for fabricating the NPA as a biocompatible chip by covalently immobilize the antibodies. The proposed nanopillar-based immunoelectrochemical biosensor exhibited good and stable electrochemical performance in β-amyloid (Aβ) detection. Moreover, we successfully confirmed the performance of the as-developed sensor using the artificial injection of Aβ in human tear, with sensitivity of 0.14 ng/mL and high reproducibility (as a standard deviation below 10%). Our findings show that the developed nanopillar-based sensor exhibits reliable electrochemical characteristics and prove its potential for application as a biosensor platform for testing at the point of care.

## Introduction

Alzheimer’s disease (AD) is an irreversible progressive brain disorder that is common among the elderly. At present, the main techniques for diagnosing AD are brain imaging from positron emission tomography and magnetic resonance imaging [1–5]. Despite the great social and economic costs of this disease, there has been slow progress in effectively treating AD, which has been exacerbated by the lack of powerful diagnostic methods. Although brain imaging is a powerful tool for diagnosing AD, intensive efforts have been made to achieve early AD diagnosis by analyzing biomarkers from biological fluid, especially from noninvasively acquired fluids such as tear and saliva. Moreover, given the relatively high protein content in tear fluid, it has become an excellent and viable candidate for the early diagnosis of AD [6–8].

Among different kinds of biomarker for AD, it was first proposed that the accumulation of β-amyloid (Aβ) peptide in brain tissue may cause neurodegeneration in AD. More recently, various research groups have reported that Aβ in ocular fluids could potentially be used as a biomarker for the diagnosis of AD [9]. In particular, low concentration of Aβ aggregates under sub-nanomolar level were discovered in parts of the eye such as the lens and retina in AD patients [10–16]. Additionally, the previous studies reveals the possibility of AD evaluation by verifying the concentration change of Aβ in the tear between healthy people with patients. Therefore, the Aβ aggregates in the tear sample could be used as a promising biomarker. The human eye is anatomically closer to the brain than other human organs; Aβ can be relatively easily obtained there without any surgical procedure. To date, few methods including enzyme-linked immunosorbent assay, mass spectrometry, surface plasmon resonance, surface-enhanced Raman spectroscopy, and nanomaterial-based imaging techniques have been developed and proposed to detect Aβ species [17–23]. Based on the affinity sensing principle with aptamer and antibody, the researches for Aβ analysis based on the minimized detection principle on biosensing chip as a point-of-care testing (POCT) device were demonstrated [24–26]. Furthermore, electrochemical biosensors have been widely utilized in the early detection of pathogens, monitoring of the quality of food and water, and clinical diagnosis due to their simplicity, sensitivity, accuracy, and rapid response [27–29]. More recently, the emergence of nanostructures in electrochemical biosensors has improved their sensitivity, response time, and portability [30, 31]. The nanopillar structure has various merits in comparison with a flat electrode in terms of electrochemical sensitivity owing to high electron transfer, enabling enhancement of the electrochemical signal. Additionally, based on its structural properties such as a high aspect ratio, a huge number of captured molecules can be stably immobilized on the nanostructure, which generates plenty of electrons; thus, we effectively obtained the increased signal via nanostructural manipulation on thin film. Based on these advantages, nanopillar structure is favorable for the analysis of biomarkers present at low levels in body fluids [30, 32].

In this paper, we propose a simple, flexible, rapid, reliable, and cost-effective electrochemical biosensor for early Aβ analysis using nanopillar-based electrodes and noninvasively acquired human tear. The thin layer of gold (Au) was deposited onto highly arrayed nanopillars to be used as working, counter, and reference electrodes. Conventionally, in previous research, Ag/AgCl was employed as a reference electrode, while gold was employed as a pseudo-reference electrode [33, 34]. The as-prepared biosensor carries out electrochemical detection in the presence of different amounts of Aβ, which is directly related to the diagnosis of AD. The performance of the electrochemical sensors was successfully confirmed by artificially injecting Aβ into human tear.

## Experimental section

### Materials and apparatus

The UV polymerizable NOA63 (Norland Optic Adhesives) was used with polyurethane (PU) (MINS-311RM, Minuta Tech.). The E-beam evaporator (EBS400) was purchased from EVATEC. The phosphate buffered saline (PBS) (#28372), 3,3′-dithiobis(sulfosuccinimidyl propionate) (DTSSP) (#21578) and bovine serum albumin (BSA) (#30063–572) were obtained from Thermo Fisher Scientific (Massachusetts, USA). HRP conjugated anti-Aβ antibody and human Aβ peptide (# 803012 and 932501) were obtained from Biolegend (California, USA). The o-phenylenediamine dihydrochloride (#p9187) was purchased from Sigma-Aldrich (Missouri, USA). DropSens (#C220AT) was obtained from PalmSens (Houten, Netherlands). The electrochemical behavior was analyzed by using the CHI 630B (CHInstruments).

### Nanopillar electrodes manipulation

The NPE was manipulated by nano-patterned mask and polymer-based flexible substrate. The stencil mask was employed to pattern the gold on the polymer substrate. To produce the nano-patterned mask, the photolithography was performed using Si wafer with PR coating. Using the nano-patterned PR, the nano-hole on the Si was modified by dry etching principle. PR-removed si was then employed as a nano-pattern mask. The polyurethane (PU) was spin-coated on the nano-hole mask, and the attached PU was cured using the UV irradiator. By releasing the PU, the flexible substrate was completed. The solidified PU was released and employed as a substrate. The titanium (Ti) and Au were deposited in regular sequence on the PU substrate employing the USB type stencil mask with soft lithography. In the 8-inch wafer, approximately 45 NPE was fabricated on a single wafer.

### Preparation of Aβ sample

The tested Aβ samples were prepared by spiking the Aβ antigen into the 0.1 M PBS buffer and artificial sample. The 1 ng/mL Aβ was firstly spiked both in PBS buffer and artificial tear sample, and the 0.1, 0.2, and 0.5 ng/mL Aβ samples were serially diluted.

### Electrochemical behavior of nanopillar electrodes

The structural properties of fabricated nanopillar arrays on the electrode was measured by employing the scanning electron microscopy (SEM). The electrochemical characterization was detected by performing the cyclic voltammetry (CV) with voltage from 0 to 0.6 V and scan rate from 10 to 500 mV/s in the 5 mM ferrocyanide. The reproducibility was investigated by scanning the 30-times CV cycling under scan rate at 10 and 50 mV/s in the 5 mM ferrocyanide. The electrochemical stability to the external pressure was tested by analyzing the CV based on the bending and twisting nanopillar electrode with 100° at least 100 times.

### Modification of chip surface for antibody immobilization

The Aβ analogue was immobilized on the NPE surface by using the 5 mM 3,3′-dithiobis(sulfosuccinimidyl propionate) in the DI water for 2 h. After washing with DI water at three times, the blocking buffer including 0.1% BSA and 0.1% triton X-100 in the PBS was applied.

### Electrochemical immuassay for Aβ analysis

Using the Aβ analogue modified NPE, the cocktail solutions of HRP-conjugated anti-Aβ antibody and Aβ antigen were reacted for 30 min. The o-phenylenediamine dihydrochloride as a HRP substrate to generate the electrochemical signal. The electric signal was evaluated by square wave voltammetry (SWV) principle, and potential was scanning from − 0.8 to 0.0 V with 0.025 V amplitude and 25 Hz frequency at 50 mV/s.

## Results and discussion

**Fig. 1:**
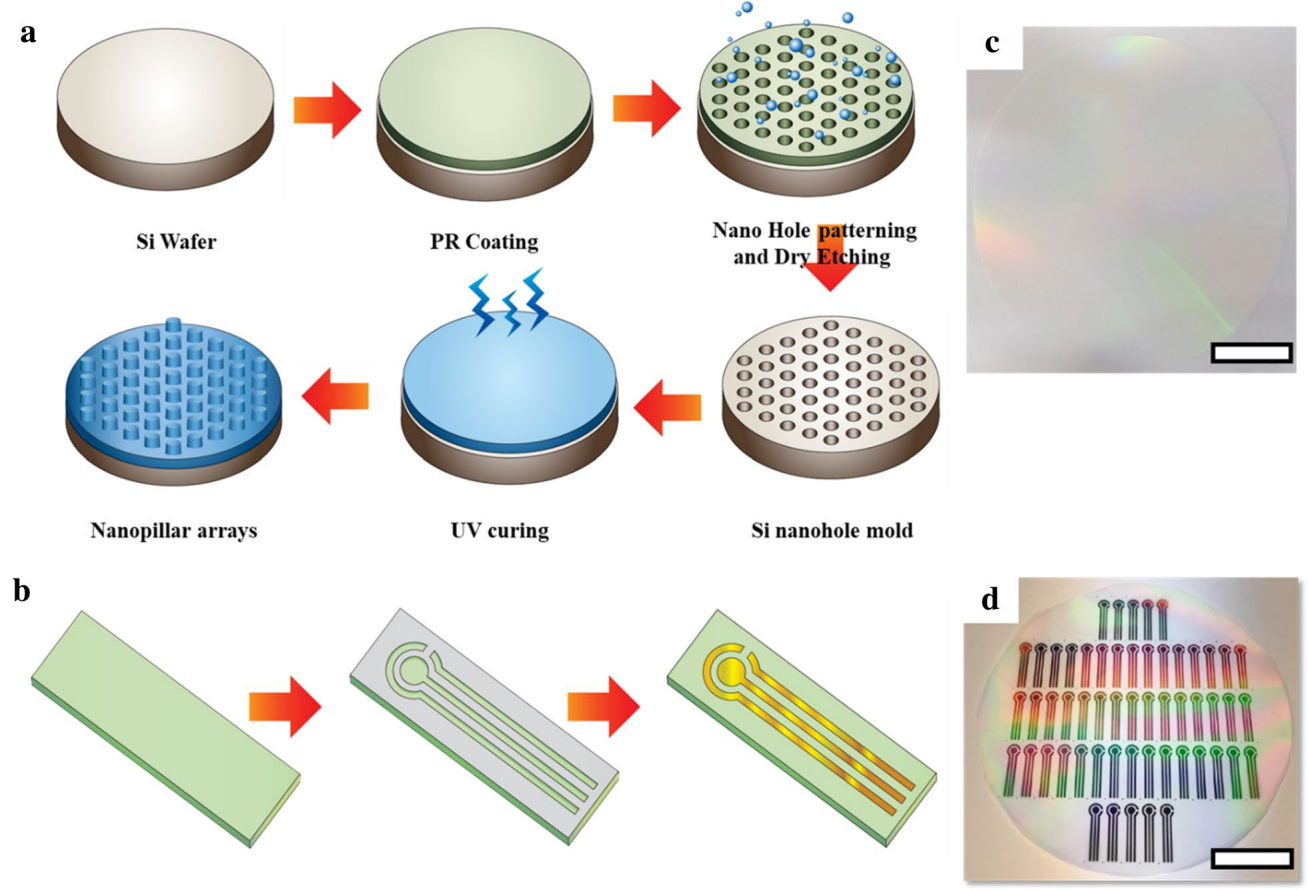
**a** Schematic illustration of fabrication process of (**a**) nanopillar arrays and (**b**) gold electrode on nanopillar arrays. Photographs of (**c**) pristine nanopillar arrays and (**d**) gold electrode on nanopillar arrays. All scale bars are 5 cm

Figures 1a, b shows the fabrication processes and product of nanopillar array (NPA) using the combination of photo- and soft lithography [35]. The NPA-based electrochemical sensors are prepared using a shadow mask for Au sputtering (Fig. 1c), and the sensor image is shown in Fig. 1d. The electrochemical sensor has a three-electrode configuration with dimensions of 32 × 8 mm^2^. When observing the sensors, the rainbow color could be observed because of light diffraction from the highly arrayed nanopillars. The electrodes are assigned as working, counting, and reference electrodes, with circular, semicircular, and rectangular shapes, respectively. The electrodes are composed of titanium (Ti) as an adhesive layer and a gold (Au) layer, which were sequentially formed onto the flexible polymeric NPA substrate using the sputtering method with the assistance of a metal-based stencil mask. Considering the portability, usability, and accessibility of the sensing platform, the sensor is specifically designed in a manner similar to the conventional USB-connectable platform. The electrochemical analysis was further carried out using a portable connector. Our findings showed that our proposed fabrication method allowed scalable, low-cost, and high-throughput fabrication.

**Fig. 2:**
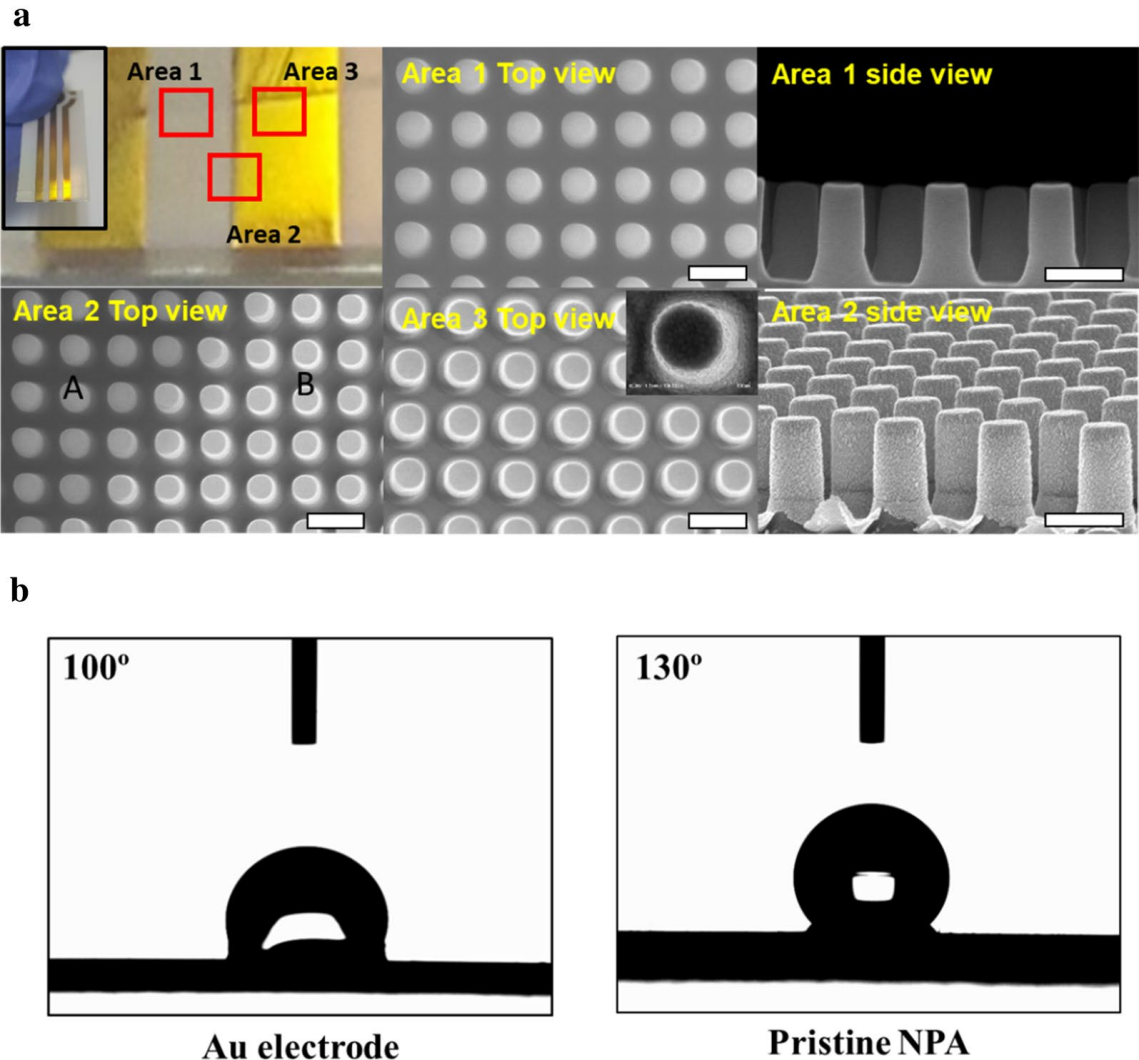
**a** Photographic images of USB-connectable NPA sensors and scanning electron microscope (SEM) images of top and side views of the indicated area. **b** contact angle (CA) of Au electrode and pristine NPA (scale bars are 2 μm)

To investigate the morphology of the sensor, different locations of the sensor surface were further investigated using SEM (Fig. 2a). The detailed dimensions of NPA are as follows: diameter of 500 nm, height of 1500 nm, and a center-to-center distance of 1000 nm; these were confirmed from the obtained SEM. The NPA was easily found without any damage, even after Ti/Au coating. Notably, the roughness of the NPA was increased upon coating with Au, and small dots were also observed from the side view of Au-coated NPA. From the obtained SEM images, there were no cracks or holes on both the Au-coated area and pristine NPA area because of strong adhesion between Au and polymeric NPA. Additionally, clear changes in contrast were found at the interface between Au-coated NPA and pristine NPA, which confirmed the successful formation of the Au electrode. The highly arrayed nanopillar provides a highly specific surface area of the electrode and facilitates the redox reaction as well as sensitivity and stability compared with the flat-surfaced sensor. To confirm the potential surface characteristic changes of the as-prepared NPA-based sensor, the changes in contact angle (CA) were observed (Fig. 2). As shown in Fig. 2b, the CA of Au-coated NPA is lower than that of pristine NPA. The Au coating enabled a decrease of the CA while enhancing the bio-friendly environment for biomarker analysis.

**Fig. 3:**
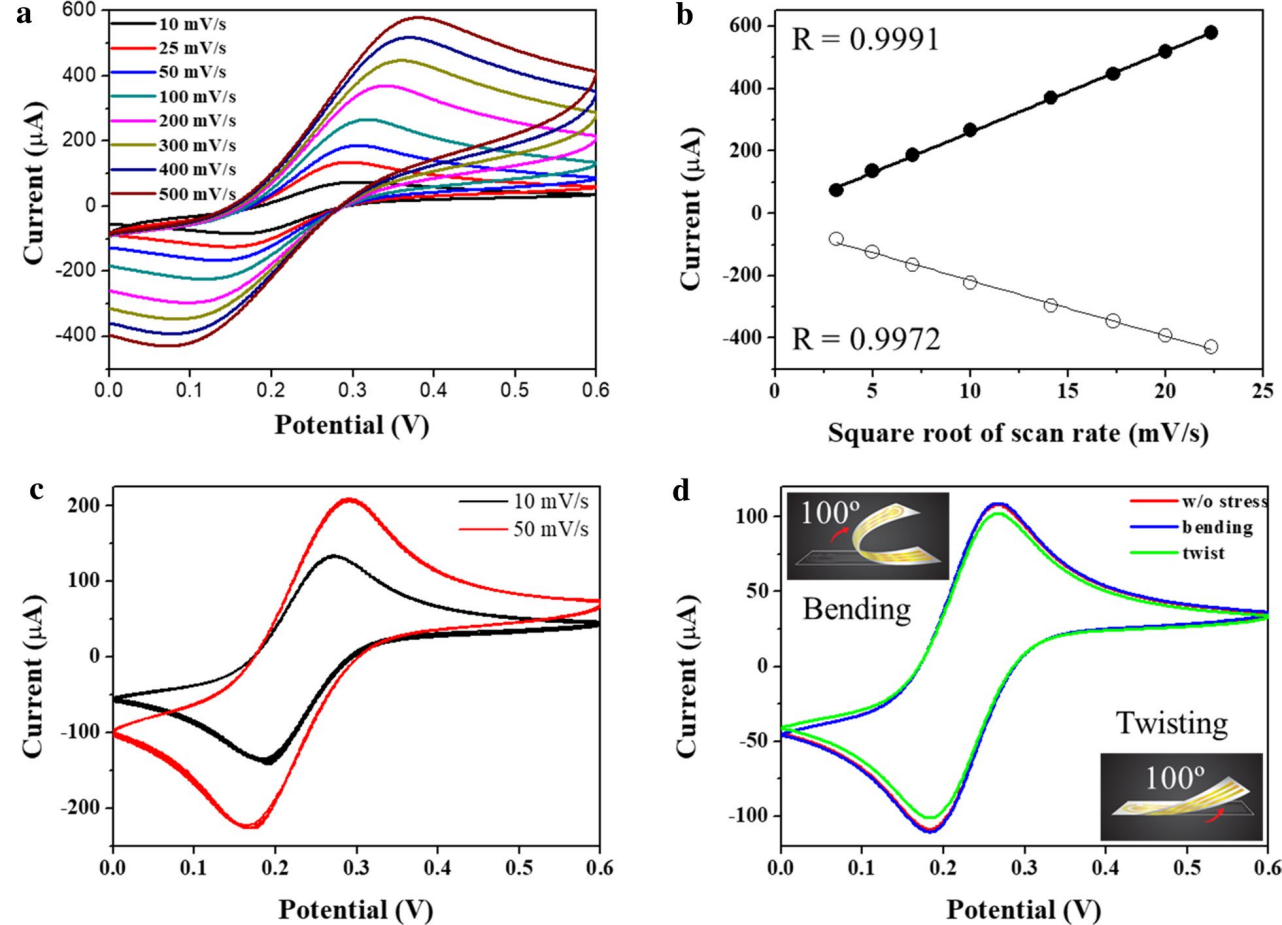
**a** Cyclic voltammogram of nanopillar electrode. The scan rate ranging from 10 to 50 mV/s was tested. **b** Redox peak current from the test result (**a**). **c** Reproducibility test of NPA at the scan rates of 10 and 50 mV/s with 30 cycles of repetition, with data from every fifth cycle presented. **d** Stability test of NPA under conditions with bending and twisting

The electrochemical characteristics of the Au electrode on NPA sensors were intensively investigated. H_2_SO_−4_ at 50 mM was used as a supporting electrolyte. Subsequently, the redox peak current changed upon increasing the scan rate from 10 to 500 mV/s without any irregular responses, as shown in Fig. 3a. The cyclic voltammetry (CV) curves of the Au-based NPA sensor displayed sharp and symmetric redox peaks. Additionally, each redox peak current’s signal was gradually increased corresponding to the scan rate. From the obtained CV results, the oxidation and calculated linearity of R^2^ values’ reduction peak currents were 0.9991 and 0.9972 across the scan range, respectively (Fig. 3b). These results suggest that the Au-based NPA electrode exhibited excellent electrochemical behavior of redox reactions.

To accurately confirm the electrochemical stability of the NPA sensors, the CV method was repeatedly performed. The electrochemical behavior was measured under 5 mM Fe(CN) with a scan rate of 10 and 50 mV; Fig. 3c presents the 30-cycle voltammogram. As shown in the figure, we acquired nearly the same graph throughout the whole detection range. Additionally, oxidation and reduction peak currents were similar to each other, indicating the high reproducibility and stability. The stability of NPA sensors against external pressure was also verified. NPA sensors were tested while bent and twisted more than 100° (inset of Fig. 3d); remarkably, the electrochemical performance was not affected. Moreover, the electrochemical behavior of redox and oxidation reactions for the bent and twisted NPA sensors was almost identical to that of the pristine NPA sensors. Considering the obtained test results, the developed NPA sensors are highly suitable for the stable analysis of biomaterials.

**Fig. 4:**
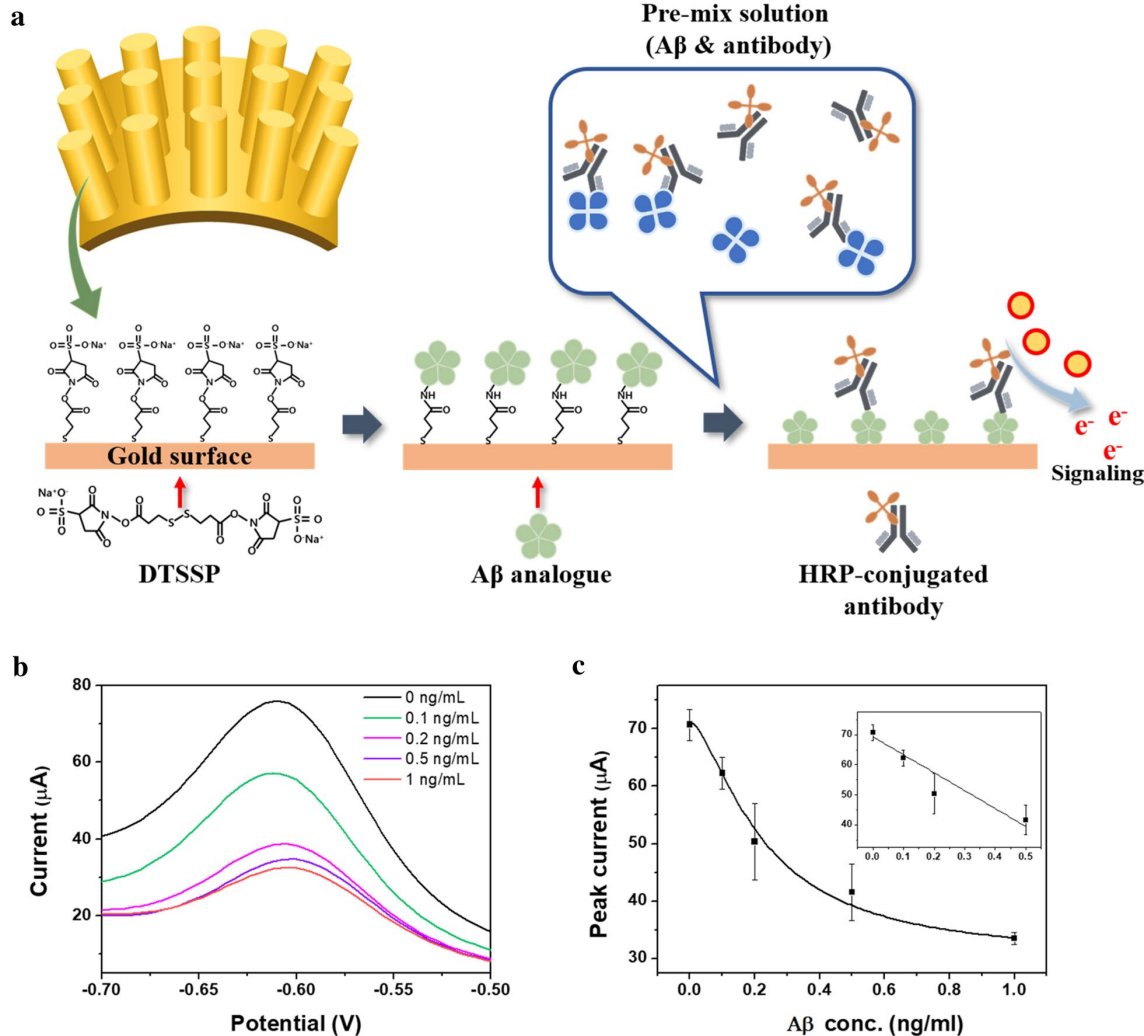
**a** Schematic illustration of electrochemical immunoassay for Aβ analysis. **b** Square wave voltammetry (SWV) graph from each Aβ in the buffer test. **c** Calibration curve of electrochemical analysis of Aβ. The linear range is presented in the inset. The error bar indicates the signal variation, and the same test was repeated three times

The Aβ concentration range from 0 to 1.0 ng/mL was analyzed using the fabricated NPA sensors. To effectively detect the Aβ, the conventional competitive immunoassay was employed and further analyzed. A mixture of prepared antigen sample and HRP-conjugated Aβ antibody was loaded on the NPA sensors, which were modified with Aβ analog. Subsequently, substrate was added to the sensors for the generation of electrochemical activity, which was measured by SWV (Fig. 4a). As shown in Fig. 4b, the obtained signal from each antigen concentration was clearly distinguished, and it was proportional to the applied Aβ concentration. The results indicated that the developed NPA structure is favorable for immunoassay-based electrochemical measurement. To clearly verify the signal change, the calibration curve was obtained by employing the peak current from each test result (Fig. 4c). The signals in the calibration curve linearly increased in accordance with the Aβ concentration. The results reveal that the antibody was effectively immobilized on the three-dimensional chip surface, and the immunoassay was successfully performed based on the nanopillar electrode. To confirm the sensitivity of the developed NPA sensors for the Aβ analysis, limit of detection (LOD) was calculated by using the standard deviation from blank and slope of regression line, and the 0.16 ng/mL was calculated. The obtained results indicated that the NPA sensors have high sensitivity based on structural characterization of NPA sensors related to effective electron delivery [36–38]. Additionally, the acquired sensitivity could be due to the high surface area of the nanostructure. For confirmation of the accuracy, the R^2^ value in the linear curve range was calculated. The recorded linearity was 0.94, meaning that the low level of Aβ could be sensitively and clearly detected with high accuracy using the NPA sensors. The coefficient of variation was also calculated to verify the reproducibility. To accurately identify the signal variation, the test was repeated at least three times under the same conditions, such as temperature, pH, concentration, and reaction time. The calculated CV in the whole detection range was 10%, indicating the high reproducibility. The calculated CV is presented in Fig. 4b as an error bar. Based on the findings with reliable test results, we successfully demonstrated that the fabricated NPA sensors could be effective for Aβ analysis with high sensitivity and accuracy.

**Fig. 5:**
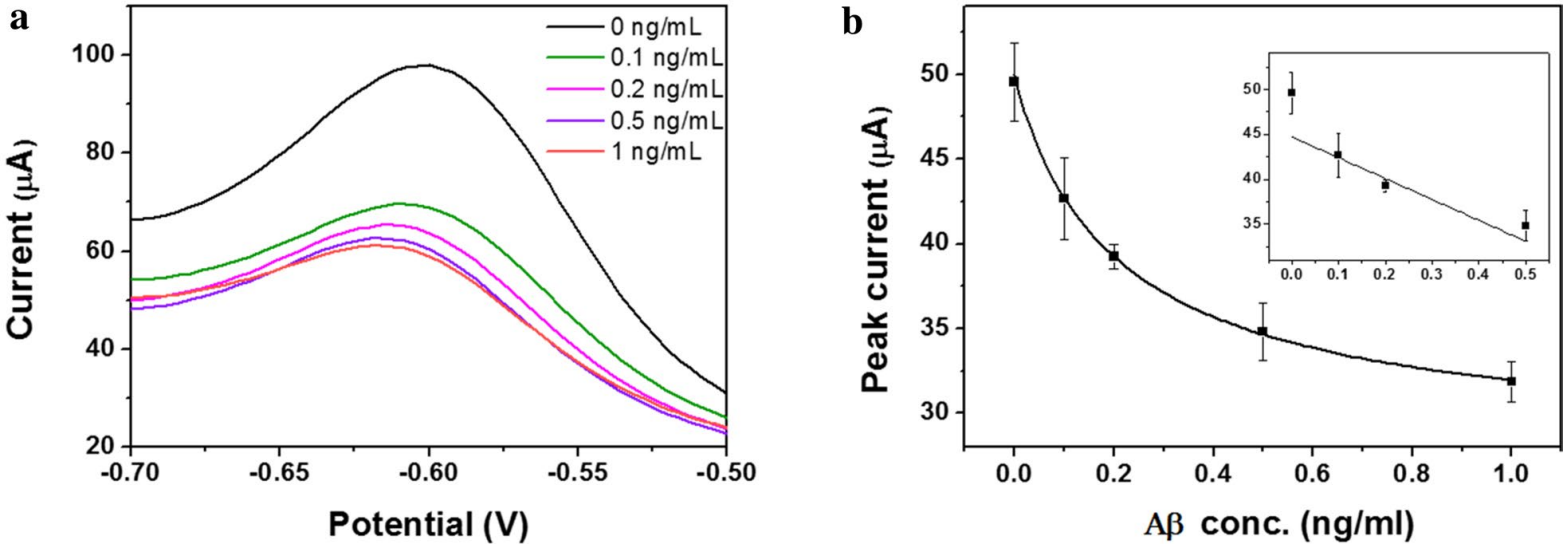
**a** The voltammogram from Aβ in the artificial tear sample. **b** Calibration curve for Aβ test. The inset indicates the linear range. The error bar indicates the signal variation, and the same test was repeated in triplicate

To verify the practical utility of the NPA sensor for Aβ measurement, an Aβ test based on actual samples was carried out by spiking various concentrations of Aβ antigen into the artificial tear sample. The Aβ concentration range from 0 to 1 ng/mL was analyzed under the same principle and conditions as in the previous buffer test using the prepared sensor chip. The electrochemical signals from each test are presented in Fig. 5a, and the signal changed in proportion to the Aβ concentration. The oxidation peak current was employed to obtain the calibration curve, as presented in Fig. 5b. The linear curve was registered in accordance with the applied Aβ concentration, which indicated that a stable electrochemical response was produced by immunoassay on the nanostructure. In comparison with the buffer test result, a slightly low signal was detected. The reason for this signal variation is that the various components in actual tear samples, such as ions, proteins, and lipids, could interfere with electron transfer on the chip surface. With regard to the use of actual original samples, the obtained signal was highly similar to the buffer test result, which proved that the developed nano-arrayed sensor shows high electrochemical performance toward Aβ in tear samples.

To verify the sensitivity, the LOD was calculated as same methods with previous formula, and the 0.14 ng/mL was obtained. The relatively high sensitivity of NPA sensors to actual Aβ is mainly attributable to the effective transfer of electrons produced by redox enzyme activity on the nanostructure. The signal variation was determined by performing repeated tests under the same conditions as in previous test; a CV value of 8% was recorded with R^2^ of 0.92, demonstrating that the developed NPA sensors could accurately and reproducibly analyze the various concentrations of Aβ. Considering the use of actual tear samples, the test results are potentially highly promising in a clinical context, in which sensitive and accurate analyses are required.

## Conclusion

In summary, this paper presents an in-depth study of a noninvasive way of detecting the Aβ associated with AD from human tear using a flexible and reliable nanopillar-based immunoelectrochemical sensor. Based on the skillful manufacturing system, we reliably fabricated the stable, reproducible, and sensitive nanopillar electrode, enabling mass production of the sensors at low cost. Additionally, using the nanostructural properties, we demonstrate the highly effective electrochemical behavior with effective biomolecule immobilization on the chip surface. The diverse range of Aβ in artificial tear confirmed the performance of the as-prepared NPA biosensor down to 0.1 ng/mL. The NPA electrodes developed in this study could provide reliable, flexible, low-cost, and disposable sensing platforms for biosensors for testing at the point of care.

## Data Availability

All the data are presented in the submitted manuscript and no supplementary information is available.

## Abbreviations

AD: Alzheimer’s disease
NPA: Nanopillar array
CV: Cyclic voltammetric
PU: Polyurethane
Aβ: β-amyloid
POCT: Point-of-care testing
Au: Gold
PBS: Phosphate buffered saline
BSA: Bovine serum albumin
Ti: Titanium
SEM: Scanning electron microscopy
CA: Contact angle
SWV: Square wave voltammetry

## References

[1] Fox NC, Black RS, Gilman S, Rossor MN, Griffith SG, Jenkins L, Koller M (2005). Neurology.

[2] Lopresti BJ, Klunk WE, Mathis CA, Hoge JA, Ziolko SK, Lu X, Meltzer CC, Schimmel K, Tsopelas ND, DeKosky ST, Price JC (2005). J. Nucl. Med..

[3] Price JC, Klunk WE, Lopresti BJ, Lu X, Hoge JA, Ziolko SK, Holt DP, Meltzer CC, DeKosky ST, Mathis CA (2005). J. Cereb. Blood Flow Metab..

[4] Stoub TR, Bulgakova M, Leurgans S, Bennett DA, Fleischman D, Turner DA, deToledo-Morrell L (2005). Neurology.

[5] Frisoni GB, Fox NC, Jack CR, Scheltens P, Thompson PM (2010). Nat. Rev. Neurol..

[6] Zhou L, Zhao SZ, Koh SK, Chen L, Vaz C, Tanavde V, Li XR, Beuerman RW (2012). J. Proteomics..

[7] Hagan S, Martin E, Enríquez-de-Salamanca A (2016). EPMA J..

[8] Aqrawi LA, Galtung HK, Vestad B, Øvstebø R, Thiede B, Rusthen S, Young A, Guerreiro EM, Utheim TP, Chen X, Utheim ØA, Palm Ø, Jensen JL (2017). Arthritis Res. Ther..

[9] Lee JC, Kim SJ, Hong S, Kim Y (2019). Exp. Mol. Med..

[10] Frederikse PH, Garland D, Jr Zigler JS, Piatigorsky J (1996). J. Biol. Chem..

[11] Goldstein LE, Muffat JA, Cherny RA, Moir RD, Ericsson MH, Huang X, Mavros C, Coccia JA, Faget KY, Fitch KA, Masters CL, Tanzi RE, Chylack LT, Bush AI (2003). Lancet.

[12] Dutescu RM, Li QX, Crowston J, Masters CL, Baird PN, Culvenor JG (2009). Clin Exp. Ophthalmol..

[13] Moncaster JA, Pineda R, Moir RD, Lu S, Burton MA, Ghosh JG, Ericsson M, Soscia SJ, Mocofanescu A, Folkerth RD, Robb RM, Kuszak JR, Clark JI, Tanzi RE, Hunter DG, Goldstein LE (2010). PLoS ONE.

[14] Koronyo-Hamaoui M, Koronyo Y, Ljubimov AV, Miller CA, Ko MK, Black KL, Schwartz M, Farkas DL (2011). NeuroImage.

[15] Koronyo Y, Salumbides BC, Black KL, Koronyo-Hanaoui M (2012). Alzheimer’s disease in the retina: imaging retinal aβ plaques for early diagnosis and therapy assessment. Neurodegener. Dis..

[16] La Morgia C, Ross-Cisneros FN, Koronyo Y, Hannibal J, Gallassi R, Cantalupo G, Sambati L, Pan BX, Tozer KR, Barboni P, Provini F, Avanzini P, Carbonelli M, Pelosi A, Chui H, Liguori R, Baruzzi A, Koronyo-Hamaoui M, Sadun AA, Carelli A (2016). Melanopsin retinal ganglion cell loss in Alzheimer disease. Ann. Neurol.

[17] Koychev I, Galna B, Zetterberg H, Lawson J, Zamboni G, Ridha BH, Rowe JB, Thomas A, Howard R, Malhotra P, Ritchie C, Lovestone S, Rochester L (2018). Aβ42/Aβ40 and Aβ42/Aβ38 ratios are associated with measures of gait variability and activities of daily living in mild Alzheimer’s disease: a pilot study. J. Alzheimers Dis..

[18] Ramakrishnan M, Kandimalla KK, Wengenack TM, Howell KG, Poduslo JF (2009). Surface plasmon resonance binding kinetics of Alzheimer’s disease amyloid β peptide-capturing and plaque-binding monoclonal antibodies. Biochemistry.

[19] Pesini P, Pérez-Grijalba V, Monleón I, Boada M, Tárraga L, Martínez-Lage P, San-José I, Sarasa M (2012). Reliable measurements of the β-amyloid pool in blood could help in the early diagnosis of AD. Int. J. Alzheimer Dis..

[20] Park SG, Ahn MS, Oh YJ, Kang M, Jeong Y, Jeong KH (2014). Nanoplasmonic biopatch for in vivo surface enhanced raman spectroscopy. BioChip J..

[21] Galozzi S, Marcus K, Barkovits K (2015). Amyloid-β as a biomarker for Alzheimer’s disease: quantification methods in body fluids. Int. J. Alzheimer Dis..

[22] Palladino P, Aura AM, Spoto G (2016). Surface plasmon resonance for the label-free detection of Alzheimer’s β-amyloid peptide aggregation. Anal. Bioanal. Chem..

[23] Shah S (2016). The nanomaterial toolkit for neuroengineering. Nano Converg..

[24] Gagni P, Sola L, Cretich M, Chiari M (2013). Development of a high-sensitivity immunoassay for amyloid-beta 1–42 using a silicon microarray platform. Biosens. Bioelectron..

[25] Gheorghiu M, David S, Polonschii C, Olaru A, Gaspar S, Bajenaru O, Popescu BO, Gheorghiu E (2014). Label free sensing platform for amyloid fibrils effect on living cells. Biosens. Bioelectron..

[26] Oh J, Yoo G, Chang YW, Kim HJ, Jose J, Kim E, Pyun JC, Yoo KH (2013). A carbon nanotube metal semiconductor field effect transistor-based biosensor for detection of amyloid-beta in human serum. Biosens. Bioelectron..

[27] Liu F, Choi KS, Park TJ, Lee SY, Seo TS (2011). Graphene-based electrochemical biosensor for pathogenic virus detection. BioChip J..

[28] Setterington EB, Alocilja EC (2012). Electrochemical biosensor for rapid and sensitive detection of magnetically extracted bacterial pathogens. Biosensors.

[29] Park YM, Lim SY, Shin SJ, Kim CH, Jeong SW, Shin SY, Bae NH, Lee SJ, Na J, Jung GY, Lee TJ (2018). A film-based integrated chip for gene amplification and electrochemical detection of pathogen causing foodborne illnesses. Anal. Chim. Acta.

[30] Park YM, Lim SY, Jeong SW, Song YS, Bae NH, Hong SB, Choi BG, Lee SJ, Lee KG (2018). Flexible nanopillar-based electrochemical sensors for genetic detection of foodborne pathogens. Nano Convergence.

[31] Seo YT, Jeong S, Lee JK, Choi HS, Kim J, Lee HY (2018). Innovations in biomedical nanoengineering: nanowell array biosensor. Nano Convergence.

[32] Yoon JH, Hong SB, Yun S-O, Lee SJ, Lee TJ, Lee KG, Choi BG (2017). High performance flexible pH sensor based on polyaniline nanopillar array electrode. J. Colloid. Interf. Sci..

[33] Hashimoto K, Ishimori Y (2001). Preliminary evaluation of electrochemical PNA array for detection of single base mismatch mutations. Lab Chip.

[34] Kim YS, Niazi JH, Gu MB (2009). Specific detection of oxytetracycline using DNA aptamer-immobilized interdigitated array electrode chip. Anal. Chim. Acta.

[35] Lee KG, Choi BG, Kim BI, Shyu T, Oh MS, Im SG, Chang S-J, Lee TJ, Kotov NA, Lee SJ (2014). Scalable nanopillar arrays with layer-by-layer patterned overt and covert images. Adv. Mater..

[36] Anandan V, Rao YL, Zhang G (2006). Nanopillar array structures for enhancing biosensing performance. Int. J. Nanomedicine.

[37] Shin C, Shin W, Hong HG (2007). Electrochemical fabrication and electrocatalytic characteristics studies of gold nanopillar array electrode (AuNPE) for development of a novel electrochemical sensor. Electrochim. Acta.

[38] Lotwala C, Ji HF (2017). Electrochemistry on nanopillared electrodes. AIMS mater. Sci..

